# Analysis of 113 Mycotoxins and Plant Toxins in Wheat, Soy and Pea Protein Concentrates from the German Market Reveals, i.a., a High Prevalence of Alterperylenol in Wheat Gluten

**DOI:** 10.3390/toxins18090377

**Published:** 2026-09-02

**Authors:** Ahmed H. El-Khatib, Arnold Bahlmann, Christoph Hutzler, Stefan Weigel

**Affiliations:** German Federal Institute for Risk Assessment (BfR), Reference Centre for Food and Feed Analysis, Max-Dohrn-Str. 8-10, 10589 Berlin, Germany

**Keywords:** QuEChERS, emerging mycotoxins, ochratoxin A, wheat gluten, pea protein, soy protein

## Abstract

Wheat, soy and pea protein isolates used for the production of plant-based meat alternatives were analyzed for 87 mycotoxins and 26 plant toxins by means of a validated QuEChERS-based LC-MS/MS multi-method. For wheat gluten, the results revealed the presence of *Alternaria* toxins, deoxynivalenol, enniatins, ergot alkaloids and ochratoxin A (OTA). For soy protein, beauvericin, enniatin B and OTA were the most frequently detected mycotoxins, while for pea protein, OTA was the most frequently detected mycotoxin. From plant toxins, the tropane alkaloids atropine and scopolamine were detected in wheat gluten and soy protein. The emerging *Alternaria* toxin alterperylenol was reported for the first time in wheat gluten (85% of samples). OTA was present at high levels in all three matrices (exceeding the EU maximum level in one wheat gluten sample). Ergot alkaloids were found in 95% of wheat gluten samples, but none exceeded the ML. The highest co-occurrence of different toxins was observed in wheat gluten (median: 22, max: 27 toxins/sample), followed by soy protein (median: five, max: nine toxins/sample) and pea protein (median: one, max: five toxins/sample).

## 1. Introduction

In recent years, plant-based meat alternatives have seen an increase in consumer popularity, with Europe having the largest share of the global meat alternative market (51.5%) [1]. The global plant protein market was estimated at $14 billion in 2024 and is expected to grow further to reach $17.4 billion by 2027 [2]. Plant-based proteins are primarily derived from cereals (e.g., wheat), legumes (e.g., pea and soybeans), oilseeds (e.g., cottonseed), and pseudocereals (e.g., amaranth and chia). Among these sources, soy protein concentrates/isolates and wheat gluten, henceforth referred to as “plant proteins”, are the most widely used ingredients in plant-based meat alternatives [1,3]. The production of plant-based proteins (>50% protein) typically involves aqueous alkaline extraction followed by protein purification through isoelectric precipitation or ultrafiltration. Alternative methods that reduce chemical usage and preserve protein quality include dry fractionation (e.g., air classification) and enzyme/ultrasound-assisted extraction [4,5]. During processing, natural toxins such as mycotoxins and plant toxins present in raw grains or legumes can be concentrated in the final products. The presence/co-occurrence of these toxins in plant-based proteins can pose potential health risks to consumers [6,7]. Several mycotoxins, such as aflatoxins, fumonisins, ochratoxin A, trichothecenes and zearalenone, are well known for their potential carcinogenicity, genotoxicity, hepatotoxicity, immunotoxicity, and/or nephrotoxicity [8,9,10]. In addition, the combined exposure to multiple mycotoxins may result in additive or synergistic toxic effects, thereby increasing potential human health risks [11]. Similarly, certain plant toxins such as pyrrolizidine alkaloids are known for their genotoxicity and hepatotoxicity and have been linked to liver tumors [12,13]. Certain populations, such as newborns and infants, are especially vulnerable to exposure to these mycotoxins and plant toxins [12,14].

Currently, aflatoxins and ochratoxin A (OTA) are regulated by the European Union (EU) in processed cereal products (containing at least 80% cereals). The maximum levels (ML) of aflatoxin B1 (AFB1) and sum of aflatoxins B1, B2, G1 and G2 in processed cereal products are 2.0 and 4.0 µg/kg, respectively. The ML of OTA in cereals and processed cereal products placed on the market for the final consumer is 3.0 µg/kg. Wheat gluten sold as an industrial ingredient not directly meant for the final consumer has a separate ML for OTA of 8.0 μg/kg. In addition, ergot alkaloids are regulated by the EU in wheat gluten. The ML of ergot alkaloids (sum) in wheat gluten is 400 µg/kg [15]. However, the presence of other toxins has also been reported [7,16]. Rodríguez-Carrasco et al. have validated a method for the simultaneous determination of 21 mycotoxins in soy-based burgers. The analysis of soy-based burger samples (n = 19) from the Italian local market revealed the presence of up to 10 different mycotoxins in 95% of samples, with alternariol monomethylether (AME) and enniatins (ENNs) being the most commonly found mycotoxins [17]. Mihalache et al. have also validated a multi-mycotoxin (n = 11) method in soy-based burger as a model matrix. The method was applied to 13 plant-based meat alternative samples based on soy, pea, chickpea, lupin, and wheat from the Italian local market. The results show that, with the exception of AFB2 and zearalenone (ZEN), all investigated mycotoxins were found at quantifiable levels, with fumonisins being the most frequently found mycotoxins [18]. The authors later applied an enhanced version of the method (n = 16 mycotoxins) to meat alternative samples based on wheat (n = 33) and legumes (n = 51) from the Italian market. The results showed that alternariol (AOH), AME, beauvericin (BEA), enniatin B (ENNB), fumonisins, tentoxin (TEN) and ZEN are the most frequently (>50%) detected mycotoxins. AFB1 was detected only in legume-based samples, and OTA was detected in 36% and 24% of wheat-based and legume-based samples, respectively [19]. Although plant toxins such as pyrrolizidine and tropane alkaloids are known to contaminate crops through accidental co-harvesting [12,20], there are no reports, to the best of our knowledge, on the occurrence of plant toxins in plant-based proteins.

Most plant-based meat alternatives marketed to the final consumer comprise many ingredients. When contaminants are found in such composite food products, the source of contamination usually cannot be identified unambiguously. While there is some data on the occurrence of mycotoxins and plant toxins in raw materials for plant proteins (e.g., pea, soybeans, soybean flour) and the final composite products containing plant proteins, such as plant-based burgers [6,7], there is no information available for the isolated plant proteins. In this work, an LC-MS/MS multi-method for the simultaneous determination of a wide range of mycotoxins and plant toxins (113 toxins; 87 mycotoxins and 26 plant toxins) in wheat, soy and pea plant proteins was developed. The method involved QuEChERS-based extraction and LC-MS/MS analysis using triple quadrupole mass spectrometry. The method was validated according to the latest EU regulations for mycotoxins [21] and plant toxins [22] in terms of recovery, precision and limit of quantification (LOQ). For regulated toxins, aflatoxins, ergot alkaloids and OTA, the method has target LOQs of ≤½ the respective MLs. The mycotoxins covered by the method include aflatoxins, *Alternaria* toxins (including the emerging toxin alterperylenol), citrinin, emerging mycotoxins (BEA, ENNs), ergot alkaloids, fumonisins, ochratoxins, trichothecenes (nivalenol and related compounds, T-2 toxin and related compounds, among others), and zearalenone and related compounds. From the plant toxins, the method includes pyrrolizidine, tropane, and quinolizidine alkaloids. The validated LC-MS/MS multi-method was applied to a total of 60 samples (20 per matrix) collected from the German market. The study was not intended as a representative survey, but rather as a first assessment of the contamination status of plant proteins. In contrast to other studies, the focus of this work was on the occurrence of toxins in the pure plant proteins to avoid other confounding factors, i.e., the presence of toxins from other ingredients (e.g., fats, spices) of meat alternatives.

## 2. Results and Discussion

### 2.1. Method Optimization

The proposed method is based on our previously validated method for milk [23], with few modifications. Briefly, the method involves QuEChERS-based extraction, defatting of the ACN supernatant with hexane, evaporation to dryness and finally reconstitution in MeOH/H_2_O and analysis by LC-MS/MS. The modifications in the proposed method include a modified order of addition of extraction and standard solutions, the extensive use of internal standards (35 IS) added before extraction or evaporation steps, and the addition of ammonia solution before evaporation (together with glycerol as a keeper) to neutralize residual formic acid.

Initial tests revealed that directly spiking wheat gluten with a small volume of multi-analyte standard solution severely affected analytical reproducibility. Apparently, the high methanol content of the stock solution (~64% MeOH) partially dissolved the gluten. When the spiked solution evaporated, a pebble-like precipitate remained, which could not be redissolved when extraction solvent was subsequently added, leading to poor recoveries and precision. These effects were not observed for soy and pea protein. Since it was not feasible to avoid evaporation of the highly volatile multi-analyte stock solution, the workflow was modified for all matrices so that the extraction solvent was added first to the sample before spiking to prevent any precipitation.

### 2.2. Method Performance and Validation

In this study, an LC-MS/MS method was developed and validated for the simultaneous determination of a wide range of mycotoxins and plant toxins in plant proteins (Table S5). The method was validated for 94, 84 and 87 toxins in wheat gluten, soy protein and pea protein, respectively, i.e., for these analytes the performance criteria specified by EU regulations were met (EU 2023/2782 and 2023/2783).

#### 2.2.1. Linearity and Range

The analytes showed good linearity, and all calibration levels fulfilled the criterion of a within ±20% deviation from the true concentrations using back-calculation. The calibration range and the equivalent working concentration range in matrix (µg analyte/kg matrix) for individual analytes are shown in Table S5.

#### 2.2.2. Recovery

**Figure 1:**
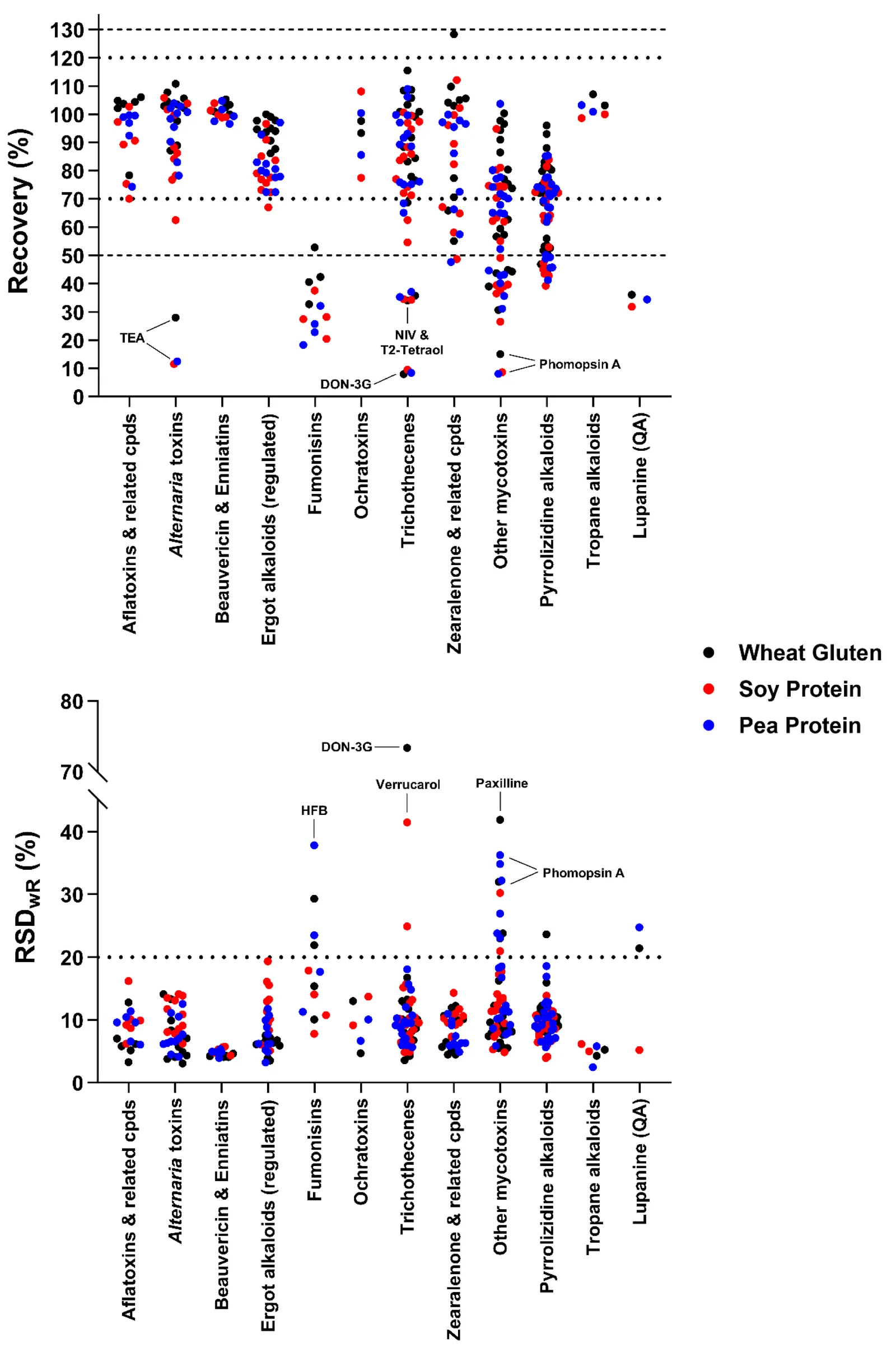
Summary of average recoveries (**top**) and within-laboratory reproducibility (intermediate precision) (RSD_wR_) (**bottom**) of extractions obtained under within-laboratory reproducibility conditions (n = 3). DON-3G: deoxynivalenol-3-glucoside; HFB: hydrolyzed fumonisin B1; NIV: nivalenol; TEA: tenuazonic acid.

The method showed sufficient recoveries for the majority of analytes in the three matrices (Figure 1). The average recoveries of individual analytes are shown in Table S5. Figure 1 shows that 86, 78 and 80% of analytes have average recoveries within the target range (50–130%) in wheat gluten, soy protein and pea protein, respectively. For analytes with average recoveries < 50%, the proposed method can still be used for screening, and the findings of these analytes in market samples in this study were used for qualitative information only. The average recoveries of 13 (12%), 18 (16%) and 17 (15%) analytes were within 30–50% in wheat gluten, soy protein and pea protein, respectively. Only three, seven and six analytes had recoveries < 30% in wheat gluten, soy protein and pea protein, respectively. Deoxynivalenol-3-glucoside (DON-3G), phomopsin A and tenuazonic acid (TEA) had the lowest recoveries in the three matrices. Other analytes with low recoveries (<30%) included dihydroergine, fumonisin B1 (FB1), fumonisin B2 (FB2), and hydrolyzed fumonisin B1 (HFB) (see Table S5).

The method development has demonstrated that the losses of analytes during extraction mainly occur during phase separation (QuEChERS) and/or evaporation steps. DON-3G, FB1, FB2, HFB, phomopsin A and TEA were shown to be lost to the aqueous phase during phase separation. Simple extraction (without QuEChERS-induced phase separation) showed acceptable recoveries for these analytes. Acid-induced cleavage does not play a significant role in the losses of DON-3G. This can be demonstrated by the acceptable recoveries for the glucoside and sulfate conjugates of AOH, AME and ZEN (IS were not used for ZEN conjugates).

#### 2.2.3. Precision

The method demonstrated excellent precision, with repeatability (RSD_r_) meeting the target criterion (≤20%) for all analytes except DON-3G and paxilline. Within-laboratory reproducibility (RSD_wR_) was found to be within the target range (≤20%) for 104 (92%), 109 (96%) and 104 (92%) of the analytes in wheat gluten, soy protein and pea protein, respectively (Figure 1). The RSD_r_ and RSD_wR_ of individual analytes are shown in Table S5. The majority of analytes that did not meet the performance criteria for RSD_r_ and RSD_wR_ were the same analytes that did not meet the performance criteria for recovery, most notably DON-3G, HFB and phomopsin A.

#### 2.2.4. Matrix Effect

**Figure 2:**
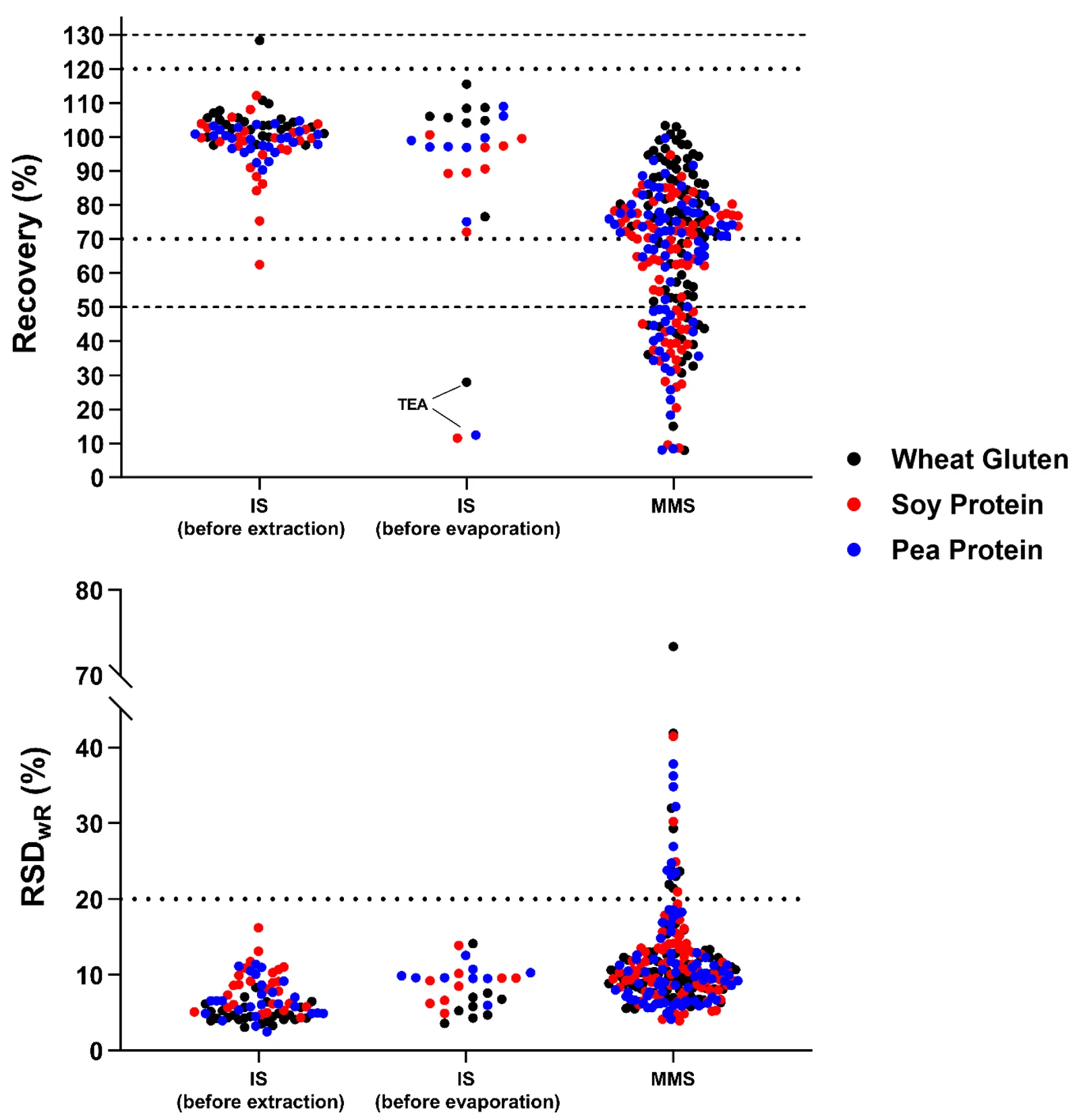
Compensation of matrix effects. Average recoveries (**top**) and within-laboratory reproducibility (RSD_wR_) (**bottom**) for all analytes in the three matrices using internal standards (IS) added before extraction, IS added before evaporation and matrix-matched standards (MMS) for the compensation of matrix effects. TEA: tenuazonic acid.

The ME caused by suppression or enhancement of analyte ionization (signal) due to the co-eluting matrix components was studied. The data for individual analytes in Table S5 show that 93 (82%), 53 (47%) and 72 (64%) analytes had an ME within the range of 100 ± 20% in wheat gluten, soy protein and pea protein, respectively. Elevated signal enhancement (ME > 120%) was demonstrated for 14, 16 and 18 analytes in wheat gluten, soy protein and pea protein, respectively. Elevated signal suppression (ME < 80%) was shown for six, 44 and 23 analytes in wheat gluten, soy protein and pea protein, respectively. To compensate for the matrix effects, a high number of internal standards (35 IS, covering the majority of the most relevant analytes) and matrix-matched calibration were implemented. Figure 2 shows that the best recoveries and precision were achieved when the IS were added before extraction (26 IS). The addition of IS before evaporation (9 IS) also improved the analytical performance (except for the recovery of TEA) compared to MMS that showed the largest number of analytes not meeting the performance criteria.

#### 2.2.5. Sensitivity

The LOD and LOQ (LVL) data for individual analytes in different matrices are shown in Table S5. To meet the ML for the regulated AFB1 and OTA in processed cereal products (2.0 and 3.0 µg/kg, respectively), the method had a target LOQ for AFB1 and OTA of ≤1.0 and 1.5 µg/kg (≤½ the ML), respectively, in wheat gluten. In addition, according to the latest regulations, each of the regulated ergot alkaloids had a target LOQ of ≤4 µg/kg in cereals and cereal-based foods [21]. Experimentally, the LOQs for aflatoxins B1, B2, G1, G2 and OTA in wheat gluten were approximately 0.5 µg/kg. The LOQs of each of the regulated ergot alkaloids (six pairs, determined as a sum) in wheat gluten were approximately 2 µg/kg. This makes the proposed multi-method suitable for the reliable determination of the regulated aflatoxins, ergot alkaloids and OTA in wheat gluten samples.

#### 2.2.6. Specificity and Carry-Over Effects

The check for false positives was included in quality assurance. Despite testing several samples from the three matrices for interfering signals, it was not possible to find a sample per each matrix that is completely free from all mycotoxins (e.g., ENNB). The samples with the least contamination were used as blanks and used for method validation. The selected HPLC column together with the careful selection of optimized MRMs (also the selection of precursor adducts), relatively long elution gradient and selection of the suitable ionization polarity resulted in a good chromatographic separation of target analytes from interfering compounds such as isomers (e.g., ergot alkaloids, pyrrolizidine alkaloids, FB2 and FB3, etc.) and/or matrix-related ions. Among the ergot alkaloids, only ergotamine and ergotaminine were not chromatographically separated and can therefore only be determined as a sum. The rest of the ergot alkaloids were well separated. With the exception of echimidine N-oxide and heliosupine N-oxide that were determined as a group, the isobaric PAs in the method were also well separated (e.g., jacobine, retrorsine and senecionine N-oxide have RT of 3.96, 6.10 and 8.83 min, respectively). HPLC carry-over between consecutive HPLC runs and the presence of analyte signals in reagent and matrix blanks and in the presence of other analytes were investigated in every batch. HPLC carry-over was observed for a few analytes, most notably for fumonisins, TEA and ergot alkaloids, but none of these interferences exceeded the calculated values of the individual LODs.

#### 2.2.7. Overall Performance of the Multi-Method

The proposed LC-MS/MS multi-method allows the simultaneous determination of mycotoxins and plant toxins in all three plant protein matrices, thereby considerably improving analytical efficiency and sample throughput. By employing a unified extraction procedure together with a single LC-MS/MS run for all target compounds, the multi-method allows the determination of a large number of toxins while using appropriate sample preparation time, reagent use, instrument occupancy, and overall operational expenses. Despite the high and diverse protein content of these matrices and the wide range of physicochemical properties and polarities of the target toxins, the proposed method demonstrated satisfactory performance and could meet the requirements of the current EU regulations for analytical methods for mycotoxins and plant toxins analysis for most analytes. The multi-method was successfully validated for 94 (83%), 84 (74%), 87 (77%) toxins in wheat gluten, soy protein and pea protein, respectively. All these validated analytes have recoveries within the target 50–130% and RSD_wR_ ≤ 20%. The LOQ of the regulated toxins were lower than the target LOQ of ≤½ their respective ML. In comparison to the published multi-methods for the determination of mycotoxins in related matrices, the proposed multi-method shows comparable (sometimes better) validation data (despite covering a larger number of analytes). Table 1 shows a comparison of the validation data in the proposed multi-method with published LC-MS/MS multi-methods validated in soy-based burger (n = 16 mycotoxins) [19] and soy-like matrix (38% protein, n = 43 mycotoxins) [24]. Overall, the validation data show the robustness of the multi-method and its applicability for reliable monitoring and risk assessment of mycotoxins and plant toxins in these protein-rich matrices. Compared to the methods in the literature, to the best of our knowledge, this is the first validated method for the analysis of mycotoxins and plant toxins in three pure plant protein concentrates/isolates that can be subsequently used for plant-based meat alternatives. In addition, the presented method covers the largest number of mycotoxins (87) and, for the first time, also plant toxins (26, covering tropane, pyrrolizidine and quinolizidine alkaloids) in any protein-rich plant-based matrices (e.g., plant-based burger).

### 2.3. Application to Plant Protein Samples

**Figure 3:**
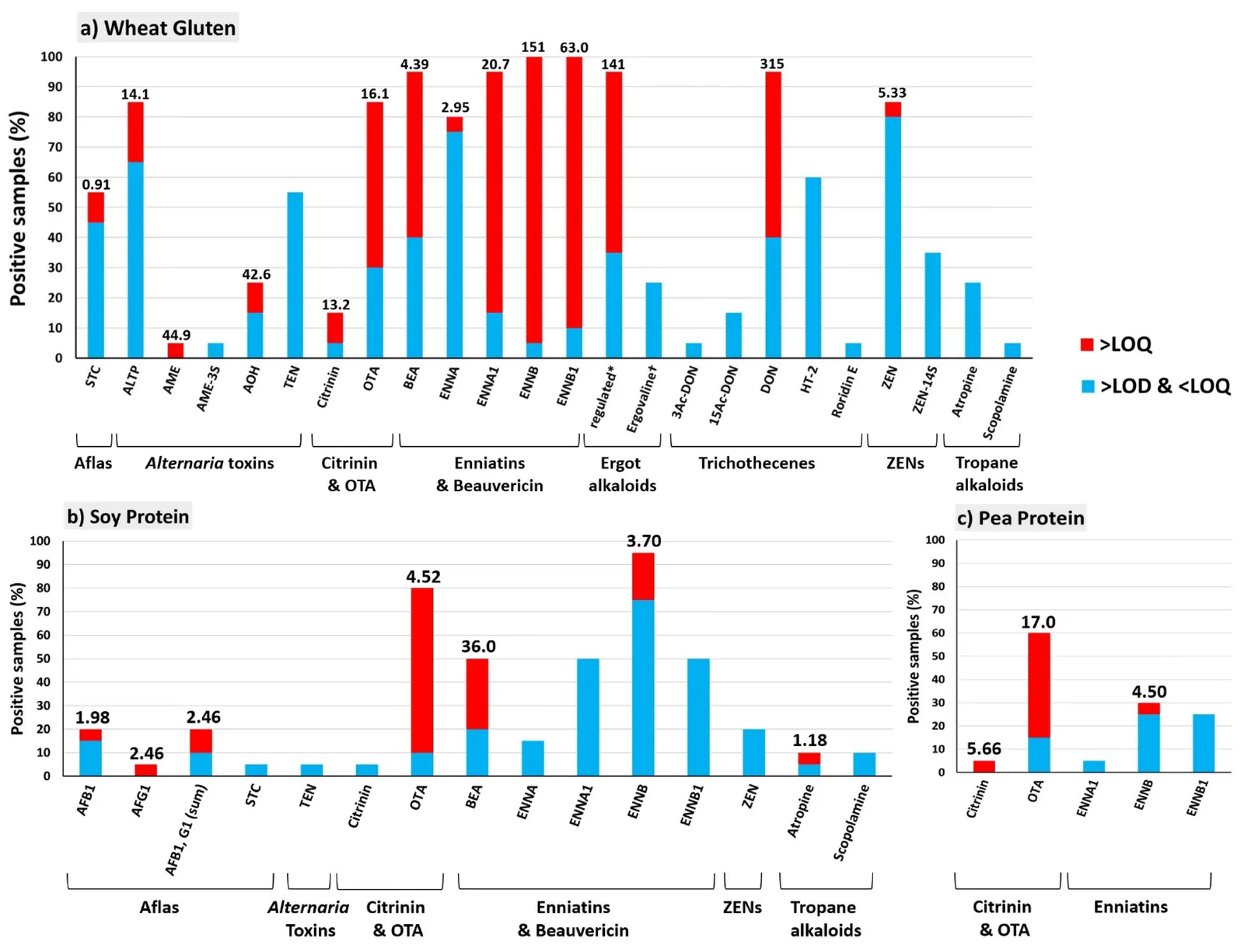
Occurrence of mycotoxins and plant toxins in the analyzed wheat gluten (**a**), soy protein (**b**) and pea protein (**c**) samples (n = 20 per matrix). The numbers on the bars indicate the maximum quantified concentrations. 3Ac-DON: 3-acetyl-deoxynivalenol; 15Ac-DON: 15-acetyl-deoxynivalenol; DON: deoxynivalenol; Aflas: aflatoxins; AFB1: aflatoxin B1; AFG1: aflatoxin G1; ALTP: alterperylenol; AME: alternariolmonomethylether; AME-3S: AME-3-sulfate; AOH: alternariol; BEA: beauvericin; ENNA: enniatin A; ENNA1: enniatin A1; ENNB: enniatin B; ENNB1: enniatin B1; ergovaline †: ergovaline + -inine (sum); OTA: ochratoxin A; regulated *: regulated ergot alkaloids (sum); STC: sterigmatocystin; TEN: tentoxin; ZENs: zearalenone (ZEN) and related compounds; ZEN-14S: ZEN-14-sulfate.

The summary of positive results (results above LOD) in the plant protein samples is shown in Figure 3, Table 2 (wheat gluten) and Table 3 (soy and pea proteins). The results of individual analytes in the respective samples and matrices are detailed in Tables S6–S8. In general, wheat gluten samples are the most contaminated in terms of the occurrence rate, the number of samples with levels exceeding LOQ, the detected toxin concentrations and the number of co-occurring toxins per sample. Soy and pea protein samples ranked second and third, respectively. The most outstanding findings were:

High levels of OTA in several samples from all three matrices.
High occurrence rate of ergot alkaloids for wheat gluten.
First reported occurrence of ALTP in wheat gluten at high occurrence rate.

#### 2.3.1. Occurrence of Regulated Mycotoxins

##### Aflatoxins

Among the aflatoxins investigated, only AFB1 and AFG1 were detected, solely in soy protein samples. In wheat gluten, the regulated aflatoxins (B1, B2, G1, G2) were not detected in any sample. In a study on wheat-based meat alternatives from Italy, only AFG1 and AFG2 were detected in one sample [19]. In soy protein, AFB1 and AFG1 were detected in four samples (20% of samples, three samples < LOQ, one sample > LOQ; 1.98 µg/kg) and one sample (5%, 2.46 µg/kg), respectively. The quantifiable levels of AFB1 and AFG1 were found in different soy protein samples (Table S7). There is no ML for aflatoxins in soy proteins. The levels of aflatoxins in our study (soy protein samples from the German market, various origins) are in agreement with previous reports. In soy protein samples from China, Singapore and USA, only AFB1 was detected in two samples (<2.01 µg/kg) [24]. In soy-based burger from Italy, only AFB1 was detected in one sample (5%, 10.1 µg/kg) [17]. In pea protein, aflatoxins were not detected in any sample. In a study on legume-based meat alternatives (e.g., soy- and pea-based) from Italy, AFB1, AFG1 and AFG2 at levels between 1.6 and 10.9 µg/kg were detected [19].

Sterigmatocystin, a precursor of AFB1 and AFG1, was detected in wheat gluten (11 samples, 55%, 9 < LOQ, 2 > LOQ; 0.859–0.910 µg/kg) and soy protein (one sample, 5%, <LOQ). In the study of Shu et al., sterigmatocystin was not detected in soy proteins [24].

It has to be noted that the other studies analyzed the processed food and not the protein isolates. Therefore, it is not possible to attribute those findings to a certain ingredient.

The production of plant-based proteins uses an aqueous alkaline extraction process. In alkaline pH, aflatoxins undergo degradation via lactone ring opening [25,26], but this depends on the reaction conditions [27]. The alkaline processing could therefore result in a reduction in aflatoxin content in the final product.

##### Ergot Alkaloids

The regulated ergot alkaloids (six pairs, determined as a sum) were found in 19 (95%, range: 2.43–141 µg/kg) wheat gluten samples. All samples were compliant with EU legislation for ergot alkaloids in wheat gluten (all < ML = 400 µg/kg). Ergocristine and ergosine were the most frequently detected ergot alkaloids (19 samples, 95%), while ergometrinine was the least frequently detected (six samples, 30%). The ergot alkaloids with the highest detected concentrations were ergotamine + ergotaminine (detected as a sum) (max: 42.6 µg/kg), ergosine (max: 23.8 µg/kg) and ergocristine (max: 21.6 µg/kg). Ergot alkaloids are known to contaminate cereals and cereal products [28]. In the production of plant-based proteins, the alkaline pH causes alkaloids to exist in the non-ionized hydrophobic free base form which may be concentrated in the final product by adsorption onto the proteins. On the other hand, depending on the reaction conditions, ergot alkaloids could be hydrolyzed in aqueous alkaline solution [29,30].

##### Ochratoxin A

High levels of OTA were quantified in all three matrices. In wheat gluten, 17 samples (85%, 6 < LOQ, 11 > LOQ, range: 0.60–16.1 µg/kg) were detected. The OTA concentration in one wheat gluten sample (16.1 µg/kg) exceeded the ML of OTA in processed cereal products (3.0 µg/kg). In soy protein, 16 samples (80%, 2 < LOQ, 14 > LOQ, 0.704–4.52 µg/kg) were detected. Among soy protein samples, the sample with the highest concentration of OTA (soy #5) was chocolate-flavored. Chocolate can contribute to the OTA finding in this sample. In pea protein, 12 samples (60%, 3 < LOQ, 9 > LOQ, 0.677–17.0 µg/kg) were detected. There is no ML for OTA in soy and pea protein concentrates/isolates. In a study on soy protein concentrates/isolates, OTA was detected in 33% of samples (range: 1.40–11.0 µg/kg) [24]. In another study on wheat-based and legume-based meat alternatives, OTA was found in 36% (range: 2.40–5.80 µg/kg) and 24% (2.10–4.83 µg/kg) of samples, respectively [19]. OTA is known to contaminate cereals, cereal products and chocolate [31]. During washing and wet milling, the less water-soluble toxins such as OTA can be concentrated in the gluten fraction [32]. However, the alkaline pH can also lead to the hydrolysis of OTA [33].

#### 2.3.2. Occurrence of Other Mycotoxins

##### *Alternaria* Toxins

*Alternaria* toxins were mainly found in wheat gluten samples. Soy protein had only one sample with a tentoxin (TEN) level below LOQ, and none was detected in pea protein. The most frequently detected *Alternaria* toxins in wheat gluten were the emerging toxin alterperylenol (ALTP) and TEN with occurrence rates of 85% and 55%, respectively. To the best of our knowledge, this is the first report on the occurrence of ALTP in wheat gluten. The ALTP findings were confirmed by high-resolution mass spectrometry (Figure S1). The *Alternaria* toxins with the highest concentrations were AME (44.9 µg/kg) and AOH (42.6 µg/kg), both found in the same wheat gluten sample. In wheat gluten, the *Alternaria* toxins were detected as follows: AOH in five samples (25%, 3 < LOQ, 2 > LOQ, range: 1.30–42.6 µg/kg), AME in one sample (5%, 44.9 µg/kg), AME-3-sulfate in one sample (5%, <LOQ), ALTP in 17 samples (85%, 13 < LOQ, 4 > LOQ, 6.46–14.1 µg/kg) and TEN in 11 samples (55%, all < LOQ). Tenuazonic acid (TEA) was detected in one sample (5%, 38.9 µg/kg, qualitative information only because the recovery of TEA was <50%), and the signal of its isomer allo-TEA was also observed (Figure S2). In wheat-based meat alternatives, AOH, AME and TEN were previously reported in 70% (4.10–38.6 µg/kg), 85% (3.20–5.95 µg/kg) and 52% (0.96–8.60 µg/kg) of samples, respectively [19]. ALTP was also reported in wheat flour in 36% (<LOQ—47.9 µg/kg) of samples [34] but was not previously reported in wheat gluten. The toxicity of ALTP was recently demonstrated [35]. It can be noted that sample gluten #13 contained the highest levels of AOH, AME and TEA (42.6, 44.9 and 38.9 µg/kg, respectively) and 8.81 µg/kg of ALTP. However, the sample with the highest level of ALTP (gluten #12, 14.1 µg/kg) contained only a relatively low level of AOH (1.3 µg/kg). In another sample (gluten #8, 12.9 µg/kg ALTP), no other *Alternaria* toxin was detected (Table S6). The difference in *Alternaria* toxins profiles in different wheat gluten samples suggests that different *Alternaria* species are involved. In a study by Zwickel et al., different *Alternaria* species were isolated, incubated and analyzed for the produced toxins. The results showed that *A. alternata, A. arborescens and A. tenuissima* species produce mainly AOH, AME and TEA and relatively few ALTP. On the other hand, strains of *A. infectoria* were characterized by producing significantly lower AOH, AME and TEA levels and relatively higher levels of ALTP [36]. The data point to the necessity to monitor wheat gluten products for *Alternaria* toxins, in particular alterperylenol.

##### Citrinin

Citrinin was detected occasionally in all three matrices. In wheat gluten, citrinin was detected in three samples (15%, 1 < LOQ, 2 > LOQ, range: 1.69–13.2 µg/kg). Furthermore, it was found in one soy protein sample (5%, <LOQ) and one pea protein sample (5%, 5.66 µg/kg). The samples that contained the highest OTA concentrations showed also the highest levels of citrinin. The wheat gluten sample (gluten #16) that had the max amount of OTA (16.1 µg/kg) had also the max amount of citrinin (13.2 µg/kg). Also, the pea protein sample (pea #19) that had the max amount of OTA (17.0 µg/kg) had also the max amount of citrinin (5.66 µg/kg). A positive correlation between OTA and citrinin concentrations in maize and wheat has been previously reported [37,38]. The alkaline pH can lead to the decomposition of citrinin [39].

##### Enniatins and Beauvericin

Enniatins (ENNs, especially ENNB) were detected in all three matrices, while beauvericin (BEA) was detected only in wheat gluten and soy protein. ENNB was the most frequently detected mycotoxin, with occurrence rates of 100%, 95% and 30% in wheat gluten, soy and pea protein samples, respectively. The highest level of emerging mycotoxins found in this study was 151 µg/kg of ENNB in a wheat gluten sample. Generally, wheat gluten had the highest occurrence rate (80–100%) of BEA and ENNs among the three matrices and also the largest detected levels of ENNs. On the other hand, the largest detected level of BEA was found in soy protein. The lowest occurrence rate was found in pea protein.

In wheat gluten, BEA and ENNB were detected in 19 samples (95%, range: 2.07–4.39 µg/kg) and 20 samples (100%, 3.26–151 µg/kg), respectively. Other ENNs were also detected at levels smaller than ENNB (Table 2). In wheat-based meat alternatives, BEA and ENNB were previously reported in 97% (5.60–36.2 µg/kg) and 94% (2.07–29.1 µg/kg) of samples, respectively [19]. In soy protein, BEA and ENNB were found in 10 samples (50%, 2.76–36.0 µg/kg) and 19 samples (95%, 2.18–3.70 µg/kg), respectively. Below-LOQ levels of ENNA, ENNA1 and ENNB1 were detected in 15%, 50% and 50% of samples, respectively. In the study on soy-based burger, ENNA (32% of samples, 3.9–633 µg/kg), ENNA1 (37%, 57.4–82.0 µg/kg), ENNB (84%, 149–178 µg/kg) and ENNB1 (63%, 62.4–276 µg/kg) were detected [17]. In pea protein, ENNB was detected in six samples (30%, 1 > LOQ; 4.50 µg/kg). ENNA1 and ENNB1 were detected at below-LOQ levels (Table 3), and BEA was not detected in pea protein samples. In legume-based meat alternatives, BEA and ENNB were previously reported in 100% (5.50–13.0 µg/kg) and 98% (2.10–59.5 µg/kg) of samples, respectively [19].

It can be noted that, despite the common co-occurrence, the levels of BEA and ENNs (especially ENNB) in samples are not necessarily correlated. In wheat gluten, the sample (gluten #19) with the highest ENNB level (151 µg/kg) has a BEA level of 2.07 µg/kg, and the sample (gluten #7) with the highest BEA level (4.39 µg/kg) has an ENNB level of 40.2 µg/kg. In soy protein, the samples with the highest BEA levels (36.0 and 12.7 in samples soy #12 and soy #20, respectively) have ENNB at below-LOQ levels. This independent occurrence of BEA and ENNs was previously reported. Yoshinari et al. have shown that BEA was found in 34% of corn grits samples, while ENNs were not detected [40].

BEA and ENNs are known to contaminate cereals and legumes [41]. Despite their poor solubility in water, the solubility of BEA and ENNs increases in alkaline pH [32,42] and could result in the reduction in BEA and ENNs in the final product.

##### Trichothecenes

Mycotoxins from the trichothecene family were only detected in wheat gluten samples, with DON being the most frequently detected mycotoxin. DON and its 3- and 15-acetylated forms were detected in 19 samples (95%, 8 < LOQ, 11 > LOQ, 41.6–315 µg/kg), one sample (5%, <LOQ) and three samples (15%, all < LOQ), respectively. HT-2 toxin and roridin E were also detected in 12 samples (60%, all < LOQ) and one sample (5%, <LOQ), respectively. In wheat-based meat alternatives, DON and HT-2 toxin were previously reported in 36% (range: 25.5–89.1 µg/kg) and 12% (12.8–93.6 µg/kg) of samples, respectively [19]. DON is one of the most common contaminants of cereals and cereal products [43]. Due to the high water solubility of DON, a major fraction of DON in the raw material is expected to be removed during the production of plant-based proteins [27,32].

##### Zearalenone and Related Compounds

Zearalenone (ZEN) was detected in wheat gluten and soy protein in 17 samples (85%, 16 < LOQ, 1 > LOQ; 5.33 µg/kg) and four samples (20%, all < LOQ), respectively. In addition, zearalenone-14-sulfate was detected in wheat gluten in seven samples (35%, all < LOQ). In soy protein, ZEN was previously reported in 17% of samples (range: 2.23–9.06 µg/kg) [24]. In wheat-based and legume-based meat alternatives, ZEN was found in 52% (range: 5.30–19.4 µg/kg) and 65% (5.30–10.6 µg/kg) of samples, respectively [19]. ZEN is commonly found in cereals [44,45]. Although ZEN is barely soluble in water, it is readily soluble in alkaline solutions. Therefore, the production of plant-based proteins using aqueous alkaline extraction could result in a reduction in ZEN content in the final product [27].

#### 2.3.3. Occurrence of Plant Toxins

The tropane alkaloids (TA) atropine and scopolamine were found in wheat gluten and soy protein samples. In wheat gluten, atropine and scopolamine were detected in five samples (25%, all < LOQ) and one sample (5%, <LOQ), respectively. In soy protein, atropine and scopolamine were detected in two samples (10%, 1 < LOQ, 1 > LOQ; 1.18 µg/kg) and two samples (10%, all < LOQ), respectively. In addition, the pyrrolizidine alkaloid intermedine (and/or its co-eluting isomers not included in the method) was detected in three wheat gluten samples (15%, 0.349–0.626 µg/kg, qualitative information only because the recovery of intermedine was <50%). To the best of our knowledge, this is the first report on the presence of plant toxins in wheat gluten and soy proteins. Seeds and plant sap of TA-containing plants are known to accidentally contaminate cereals and soybean [20]. In a recent study, a reduction in levels of atropine and scopolamine in tofu compared to the raw soybeans was demonstrated. The majority of atropine and scopolamine were found in soak water [46]. The fate of TAs should be investigated under the conditions used for the production of plant-based proteins, considering that atropine undergoes degradation under alkaline conditions [47].

#### 2.3.4. Co-Occurrence of Toxins

**Figure 4:**
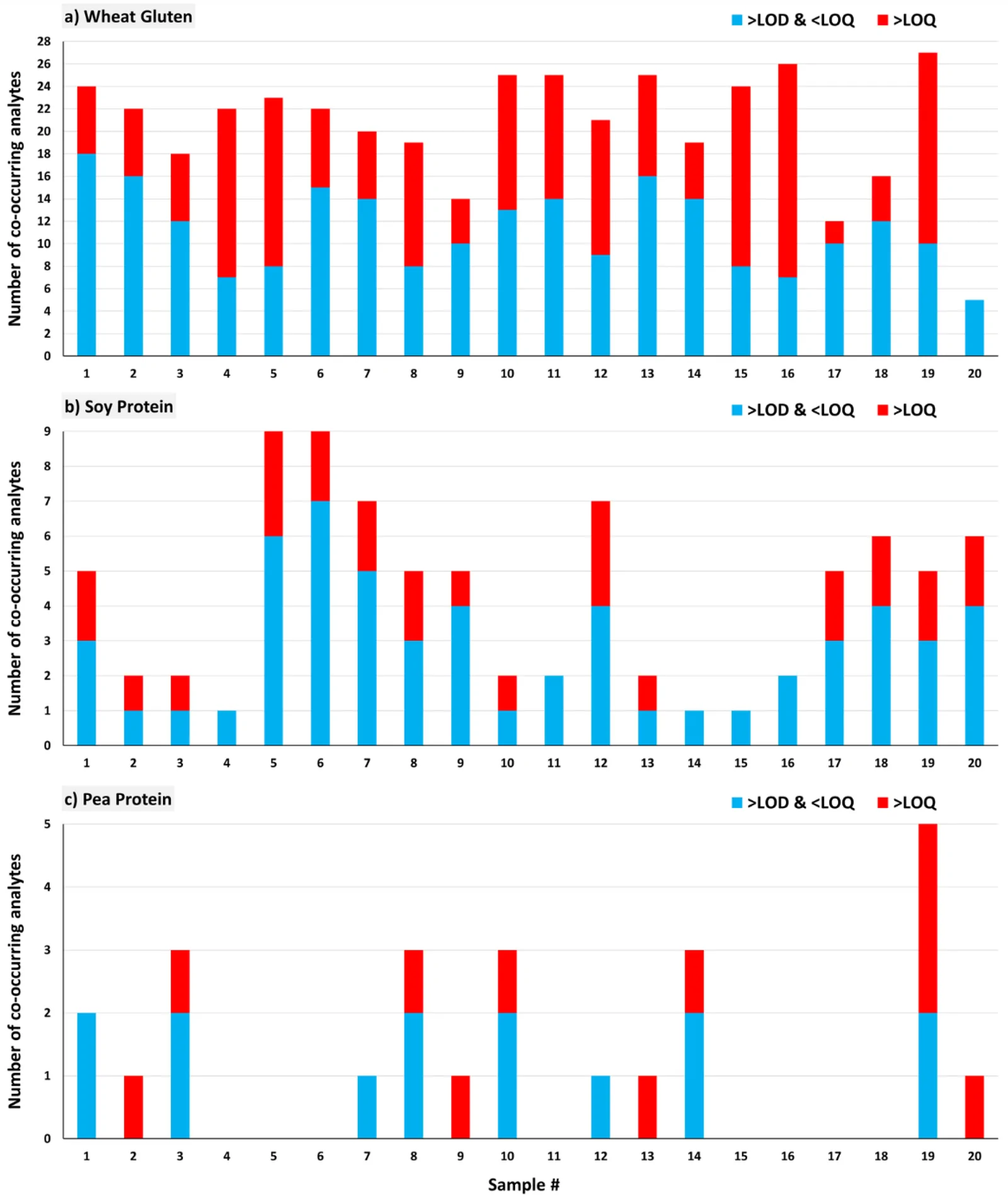
Co-occurrence of mycotoxins and plant toxins in the analyzed wheat gluten (**a**), soy protein (**b**) and pea protein (**c**) samples (n = 20 per matrix).

The findings showed the co-occurrence of multiple toxins in the samples and also demonstrated the different patterns of contamination in the three matrices (Figure 4). In wheat gluten and pea protein, samples that had the max amount of OTA had also the max amount of citrinin. Similarly, the highest concentrations of AOH and AME were found in the same wheat gluten sample. Wheat gluten samples have the highest number of co-occurring toxins per sample (range: 5–27 toxins, median: 22 toxins) followed by soy protein (1–9 toxins, median: five toxins) and finally pea protein (0–5 toxins, median: one toxin). Wheat gluten samples have also shown the highest number of co-occurring toxins with levels > LOQ per sample (0–19 toxins, median: eight toxins), followed by soy protein (0–3 toxins, median: two toxins) and pea protein (0–3 toxins, median: zero). Samples with no detected toxins were only found in pea proteins (eight samples, 40%). In general, the emerging mycotoxins ENNs and OTA were the most co-occurring toxins among the three matrices. Specifically, in wheat gluten, ALTP, BEA, DON, ENNs, ergot alkaloids, OTA and ZEN were co-occurring in 12 (60%) samples. In soy protein, BEA, ENNs and OTA were co-detected in nine (45%) samples. In pea protein, ENNB and OTA were co-detected in six (30%) samples. The co-occurrence profile in wheat gluten samples is the typical contamination profile in wheat. For example, *Alternaria* toxins, DON, ENNs and ZEN were previously shown to be the most frequently co-occurring toxins in wheat grains (ergot alkaloids and OTA were not measured) [48]. Potential differences in the contamination profiles and concentration ranges in plant-based proteins compared to raw materials could be expected due to the processing steps. Due to the lack of sufficient consumption data for this emerging food category, risk assessment could not be performed for the detected toxins in plant-based protein products. In addition, the potential health effects of chronic exposure to these toxins and their potential combined effects remain unclear and warrant additional investigation.

## 3. Conclusions

The aim of this work was to develop and validate a multi-method for the simultaneous quantitative determination of 113 toxins (87 mycotoxins and 26 plant toxins) in plant-based proteins and to investigate the (co-)occurrence of these toxins in market samples from Germany. The study focused on three pure plant proteins (wheat gluten, soy and pea proteins) that can be subsequently used for plant-based meat alternatives. The QuEChERS-based LC-MS/MS multi-method was validated according to the latest EU regulations (EU 2023/2782 and 2023/2783) and showed acceptable validation results. The method also exhibited sufficient sensitivity, and the determined LOQ levels were below the regulatory requirements for the regulated aflatoxins, ergot alkaloids and OTA. This is the first validated method for the analysis of mycotoxins and plant toxins in these pure plant proteins. In addition, the proposed method covers the largest number of mycotoxins (87) and, for the first time, also plant toxins (26) in any protein-rich plant-based matrices. The application of the validated method to market samples showed that wheat gluten samples have the highest (co-)occurrence rate (median: 22 toxins/sample), the highest number of samples with levels exceeding LOQ (19 samples, 95%) and the highest detected toxin concentrations. Soy and pea protein samples came second and third, respectively. Enniatins (especially ENNB) and OTA were the most frequently occurring toxins in the three matrices. The emerging *Alternaria* toxin alterperylenol was reported for the first time in wheat gluten (85% of samples). The results also showed high levels of OTA in all three matrices (exceeding the ML in one wheat gluten sample). Ergot alkaloids were found in 95% of wheat gluten samples, but none exceeded the ML. In addition, relatively high levels of AOH and AME were quantified in one wheat gluten sample. The plant toxins atropine, intermedine and scopolamine were detected in a few wheat gluten and soy protein samples.

In conclusion, it was demonstrated that it is highly relevant to monitor plant-based proteins and plant-based meat alternatives in which they are used for a wide range of mycotoxins and plant toxins. To this end, it is essential to have validated sensitive broad scope multi-methods available. It would be beneficial to study the fate of the toxins during the alkaline extraction of the proteins from the raw materials in order to derive processing factors and explore possible mitigation potentials.

## 4. Materials and Methods

### 4.1. Chemicals and Standards

Reference standards and isotopically labeled internal standard (IS) were purchased from the following commercial sources: Alfarma s.r.o. (Cernosice, Czech Republic), ASCA GmbH (Berlin, Germany), Biomol GmbH (Hamburg, Germany), Biopure, Romer Labs^®^ Inc. (Butzbach, Germany), Biozol GmbH (Eching, Germany), Cfm Oskar Tropitzsch GmbH (Marktredwitz, Germany), LGC Standards (Wesel, Germany), PhytoLab GmbH (Vestenbergsgreuth, Germany), Phytoplan Diehm & Neuberger GmbH (Heidelberg, Germany), Santa Cruz Biotechnology Inc. (Heidelberg, Germany), Sigma-Aldrich (Schnelldorf, Germany) and Toronto Research Chemicals (TRC, Ontario, Canada). The specific details are provided in Table S1. Acetonitrile (ACN), ammonium formate, formic acid (FA), hexane and methanol (MeOH) were of LC-MS grade and purchased from Merck (Darmstadt, Germany). Ammonia solution and glycerol were purchased from Sigma-Aldrich (Schnelldorf, Germany), and QuEChERS salts (NaCl, MgSO_4_) were purchased from Agilent Technologies (Waldbronn, Germany). Double-deionized water was obtained using a Milli-Q system from Merck (Merck Millipore, Darmstadt, Germany).

### 4.2. Plant Protein Samples

Overall, 60 plant protein samples of various origins (Austria, Canada, China, Germany, Italy, Poland, UK, USA) were purchased from the German market via online shops. Samples were selected to cover as many different suppliers as possible up to a number of 20 samples per matrix. The samples included wheat gluten (n = 20, ingredients: 100% wheat gluten), soy protein (n = 20, at least 90% soy protein) and pea protein (n = 20, 100% pea protein). All samples were stored at room temperature until analysis. The samples are listed in Table S2.

*Blank samples for method validation*: The samples were screened for the presence of mycotoxins and plant toxins using LC-MS/MS. It was not possible to find a sample completely free from all mycotoxins. The samples with the least contamination were selected as blanks and used for method validation. For wheat gluten, the blank (sample gluten #20) contained non-quantifiable traces of *Alternaria* toxins, BEA, ENNs, ergot alkaloids, and sterigmatocystin. For soy protein, the blank (sample soy #4) contained non-quantifiable traces of BEA, citrinin, and ENNs. For pea protein, the blank (sample pea #4) contained non-quantifiable traces of ENNB and sterigmatocystin. These traces in blank samples were considered in any calculations of concentrations (e.g., calculating recovery for spiked quality control (QC) samples).

### 4.3. Sample Preparation

**Figure 5:**
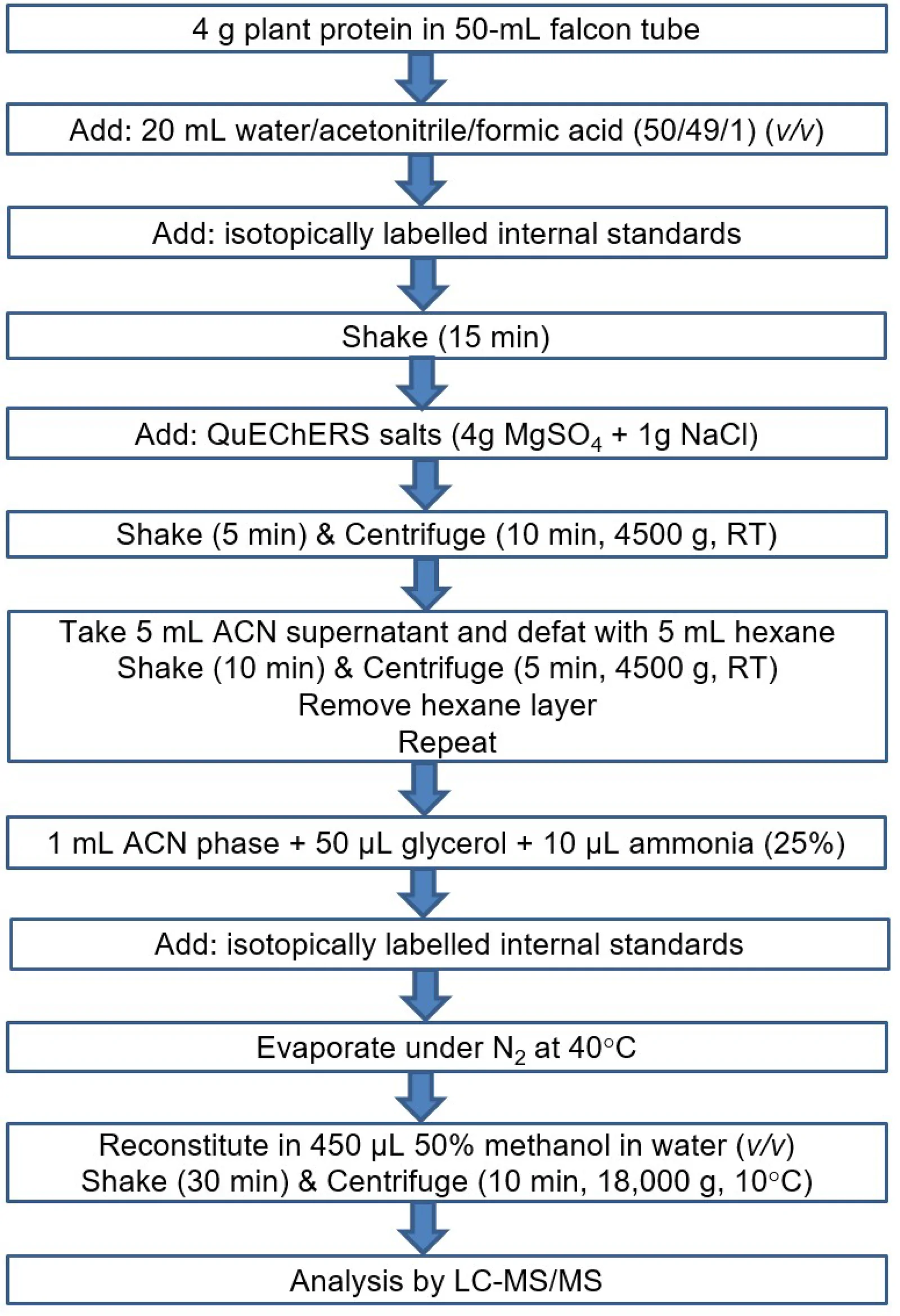
Extraction of mycotoxins and plant toxins from plant proteins.

The sample preparation includes QuEChERS-based extraction and is summarized in Figure 5. Before analysis, samples were homogenized overnight using a drum hoop mixer. For each sample, a sample intake of 4 g was prepared in duplicate, and its extract was injected twice. All samples were spiked with a total number of 35 IS, which were added either before extraction (26 IS) or before evaporation (9 IS) (details in Table S3). Apparatus: Drum hoop mixer: JEL RRM, J. Engelsmann AG (Ludwigshafen, Germany); shaker: Multi Reax, Heidolph Instruments (Schwabach, Germany); centrifuge: Heraeus Megafuge 16, Thermo Fisher Scientific (Waltham, MA, USA); concentration workstation: TurboVap LV, Caliper Life Sciences (Hopkinton, MA, USA). Samples with analyte levels exceeding the upper working range of the method were diluted and reanalyzed.

### 4.4. Stock and Working Standard Preparation

The multi-analyte stock solution was prepared by combining 22 different solutions: 11 single analyte solutions and 11 mixed solutions containing different numbers of analytes. Due to the variable solubilities of analytes, the solutions were prepared in either ACN, 50% ACN in water (*v*/*v*), MeOH or 50% MeOH in water (*v*/*v*). When stored at −20 °C, the multi-analyte stock solution was stable for at least 6 months. A working standard mixture was freshly prepared by diluting the multi-analyte stock solution to a final composition of approximately 50% MeOH in water (*v*/*v*). The concentrations of the individual standards in the multi-analyte stock solution are listed in Table S3. For calibration, a series of solutions were prepared in 50% MeOH in water (*v*/*v*) and blank extract (matrix-matched calibration) (Table S3).

### 4.5. LC-MS/MS Instrumentation and Measurements

Analyses of extracted samples were performed on a Shimadzu Nexera X2 HPLC system including binary pumps, a degasser, a column oven, an autosampler, and a control unit (Shimadzu Corporation, Duisburg, Germany) coupled to a QTrap 6500+ mass spectrometer (Sciex Germany GmbH, Darmstadt, Germany) equipped with an IonDrive™ Turbo V electrospray ionization (ESI) source. Chromatographic reversed-phase (RP) separation with 3 μL injection volume was achieved on a Waters XBridge Premier BEH C18 column (150 × 2.1 mm, 2.5 μm particle size) with guard column (Waters, Milford, MA, USA) at a flow rate of 0.35 mL/min and a column oven temperature of 40 °C. The binary mobile phase consisted of 5 mM ammonium formate and 0.1% FA in both water (eluent A) and methanol (eluent B). The gradient elution was adopted as follows: 0 min 10% B; 2 min 15% B; 9 min 33.5% B; 17 min 77.8% B; 19 min 100% B; 22 min 100% B; 22.1 min 10% B; 26 min 10% B. MS detection was conducted using positive and negative electrospray ionization (ESI) modes (polarity switching) and measuring in scheduled multiple reaction monitoring (sMRM) mode using the mass transitions and MS/MS conditions shown in Table S4. The following instrumental settings were applied: curtain gas: 40; collision gas: high; temperature: 300 °C; ion spray voltage: 4500 (−4000) V; ion source gas 1:40; ion source gas 2:40. A diverter valve cut off the flow to the MS ion source before minute 1.5 and after minute 22.5.

### 4.6. Method Validation

The method was validated to meet the performance criteria specified in the EU regulations for mycotoxins [21] and plant toxins [22] analysis. To save resources and minimize the validation workload, the principle of commodity group validation was applied. The three matrices, wheat gluten, soy protein, and pea protein, belong to the same commodity group (high starch and/or protein content and low water and fat content) [21,22]. The full method validation was performed on wheat gluten as a representative of the commodity group. For soy and pea proteins, the validity of the method was verified using limited additional validation.

*QC validation levels*: Spiking at two levels (L1, L2 (=2.5 × L1)) (n = 3 days) was conducted. A third level (L3 (=5 × L1)) was used for wheat gluten matrix to test the performance of analytes that did not meet the validation acceptance criteria at L1. The concentrations of individual toxins in the spiked samples at the 3 levels are shown in Table S3.

*Full method validation (wheat gluten)*: Day 1 (6 QC replicates/level), days 2 and 3 (one QC/level). *Verification (soy and pea proteins)*: Day 1 (2 QC replicates/level), days 2 and 3 (one QC/level) [49].

The method validation parameters were determined as follows:

− *Linearity and Range:* A series of matrix-matched standard (MMS) solutions (6 calibration levels) in the ranges listed in Table S3 were evaluated.
− *Recovery:* Average recovery of QC samples from different days.
− *Precision:* Repeatability (intra-day precision, RSD_r_) and within-laboratory reproducibility (inter-day precision, RSD_wR_) were determined for the QC samples from day 1 (RSD_r_) and all days (RSD_wR_).
− *Limit of Quantification (LOQ):* For official control purposes (checking compliance against maximum, guidance or indicative levels), the EU regulations 2023/2782 and 2023/2783 define the LOQ as the lowest successfully validated level (LVL) which is “the lowest tested concentration of analyte in a sample material, for which it has been demonstrated that the criteria for recovery, precision, and identification are met”. In this study, LOQ = LVL. The target lowest spiked concentrations of the regulated toxins, aflatoxins, ergot alkaloids and OTA, were ≤½ the respective MLs.
− *Limit of Detection (LOD):* For risk assessment and monitoring purposes, reporting a fit-for-purpose LOD level is acceptable to help generate numerical data for the majority of samples analyzed to avoid left-censored data and hence perform accurate exposure assessment. For this purpose, LOD was determined using a modified approach based on the EURL Guidance Document on the Estimation of LOD and LOQ for Measurements in the Field of Contaminants in Feed and Food [50] using the standard deviation of spiked blank samples. For wheat gluten, the sufficient number of eight replicates spiked at L1 were used to calculate the “wheat gluten LOD”. For soy and pea protein, the total number of 16 blank samples spiked at L1 from all three matrices was used to calculate the “commodity LOD”.
− *Matrix Effect (ME):* Ratio of the response of the MMS solutions to the response of standard solutions prepared in 50% MeOH in water (*v*/*v*).
− *Specificity and Carry-Over Effects:* The presence of interfering signals with MRM transitions and/or the presence of mycotoxins and plant toxins in non-spiked extracts were tested in several samples. The chromatographic separation (including testing several columns and LC gradients) and the selection of interference-free MRMs were investigated and optimized during method validation using spiked extracts to ensure good chromatographic separation of target analytes from interfering compounds such as isomers or matrix-related ions. It has to be noted that, due to interference of AFB1-d_3_ in soy, the average recovery of AFB1 in soy was calculated using MMS. HPLC carry-over was estimated by injecting a solvent blank after the highest calibration level. In the analytical batch, 2 solvent blanks were injected after the highest calibration level, and a blank was injected every 10 sample injections. Reagent blank, blank matrix extract and QC samples were injected at the beginning, middle and the end of the batch.

To externally verify its accuracy, the method was applied in a proficiency test for mycotoxins in baby food and compound feed. For all 24 parameters assessed, excellent z-scores (|z| ≤ 2) were obtained, confirming the reliability of the method in general (Table S9).

### 4.7. Data Analysis

LC-MS/MS data evaluation was performed with MultiQuant Software, ver. 3.0.2 (AB Sciex Germany GmbH, Darmstadt, Germany).

### 4.8. Identification

The retention time of the analyte in the extract should match that of the calibration standard solutions with a tolerance of ±0.1 min. Peaks of both MRM transitions in the extracted ion chromatograms must fully overlap. The ion ratio of MRM transitions from sample extracts should be within ±30% of average of the ion ratios of the calibration standards from the same sequence. Hereafter, a sample is considered positive for a certain analyte when the identification criteria are met in all four injections of the prepared sample.

## Figures and Tables

**Table 1:** Comparison of validation parameters of the proposed multi-method with published multi-methods in plant protein-rich matrices.

	Proposed Method									[19]			[24]		
Matrix	Wheat Gluten			Soy Protein			Pea Protein			Soy-Based Burger			Soy-Like Matrix (38% Protein)		
Parameter	Recovery	RSD_wR_	LOQ *	Recovery	RSD_wR_	LOQ *	Recovery	RSD_wR_	LOQ *	Recovery	RSD_wR_	LOQ ^$^	Recovery	RSD_wR_	LOQ ^§^
	(%)	(%)	[µg/kg]	(%)	(%)	[µg/kg]	(%)	(%)	[µg/kg]	(%)	(%)	[µg/kg]	(%)	(%)	[µg/kg]
Aflatoxin B1	104	5.1	0.52	75	16	1.3	99	6.1	0.52	76	6	0.97	80	2.5	2.0
Aflatoxin B2	102	6.1	0.52	97	9.9	0.52	92	11	0.52	74	3	0.95	91	3.9	0.97
Aflatoxin G1	106	7.0	0.51	89	9.2	0.51	97	9.6	0.51	81	6	1.31	83	3.4	1.1
Aflatoxin G2	104	3.3	0.52	103	8.7	0.52	100	6.6	0.52	82	2	1.51	85	4.5	0.62
Alternariol	108	4.9	5.3	106	8.6	5.3	102	6.6	5.3	83	5	0.92	108	5.9	12
Alternariolmetyhlether	102	3.8	5.3	88	11	5.3	95	6.2	5.3	81	2	0.96	108	2.2	2.3
Beauvericin	105	4.3	2.1	104	5.7	2.1	105	3.9	2.1	80	9	0.03	109	9.6	16
Deoxynivalenol	77	4.3	26	72	4.9	26	75	6.0	26	91	7	3.59	87	7.8	2.4
Enniatin B	102	4.6	2.1	99	5.3	2.1	99	4.9	2.1	79	2	0.05	67	5.8	7.2
HT-2 toxin	109	7.6	5.2	101	10	5.2	100	10	5.2	80	12	1.21	-	-	-
Ochratoxin	98	4.7	0.54	108	9.1	0.54	100	10	0.54	81	6	0.42	96	2.7	1.3
T-2 toxin	115	6.8	5.1	100	6.6	5.1	109	9.5	5.1	82	4	0.30	-	-	-
Tentoxin	111	4.3	5.2	102	12	5.2	103	7.0	5.2	88	7	0.38	104	17	3.8
Zearalenone	110	4.5	5.1	112	7.3	5.1	100	5.8	5.1	98	12	0.09	105	2.3	2.2

* LOQ = LVL (lowest validated level); ^$^ LOQ determined by signal-to-noise ratio method; ^§^ LOQ determined by calibration curve intercept variance method.

**Table 2:** Occurrence of mycotoxins and plant toxins in the analyzed wheat gluten samples from Germany (only validated analytes with at least one sample > LOD are shown).

	Wheat Gluten (n = 20)										
Analyte	LOQ [µg/kg]	Positive Samples (>LOD)		# Samples >LOD & <LOQ	# Samples > LOQ	Max ^$^ [µg/kg]	Range ^$^ [µg/kg]	Lower Bound [µg/kg]		Upper Bound [µg/kg]	
		#	Rate (%)					Median	95th Percentile	Median	95th Percentile
**Aflatoxins**											
Sterigmatocystin	0.531	11	55	9	2	0.910	0.859–0.910	0.095	0.862	0.507	0.862
***Alternaria*** **toxins**											
Alternariol	5.32	5	25	3	2	42.6	1.30–42.6	-	3.36	1.30	6.82
Alternariolmetyhlether	5.28	1	5	-	1	44.9	-	-	2.25	0.900	3.10
Alternariolmetyhlether-3-Sulfate	5.15	1	5	1	-	-	-	-	0.076	1.52	1.68
Alterperylenol	5.16	17	85	13	4	14.1	6.46–14.1	2.14	13.0	5.80	13.0
Tentoxin	5.17	11	55	11	-	-	-	0.668	0.668	4.67	4.67
**Citrinin and Ochratoxin A**											
Citrinin	5.15	3	15	1	2	13.2	1.69–13.2	-	2.27	1.69	5.54
Ochratoxin A	0.541	17	85	6	11	16.1	0.60–16.1	0.605	2.47	0.605	2.47
**Enniatins and Beauvericin**											
Beauvericin	2.07	19	95	8	11	4.39	2.07–4.39	2.24	4.28	2.24	4.28
Enniatin A	2.06	16	80	15	1	2.95	-	0.400	0.528	2.06	2.11
Enniatin A1	2.08	19	95	3	16	20.7	2.12–20.7	4.76	9.78	4.76	9.78
Enniatin B	2.08	20	100	1	19	151	3.26–151	37.8	131	37.8	131
Enniatin B1	2.08	20	100	2	18	63.0	5.20–63.0	15.9	35.6	15.9	35.6
**Ergot alkaloids (EA)**											
Regulated EA (sum)	2.07–2.31	19	95	7	12	141	2.43–141	8.82	126	25.9	129
Ergovaline + -inine (sum)	2.07	5	25	5	-	-	-	-	0.559	0.559	2.57
**Trichothecenes**											
3-Acetyl-Deoxynivalenol	5.14	1	5	1	-	-	-	-	0.038	0.765	0.964
15-Acetyl-Deoxynivalenol	5.14	3	15	3	-	-	-	-	1.32	1.32	4.86
Deoxynivalenol	25.6	19	95	8	11	315	41.6–315	42.1	254	42.1	254
HT-2-Toxin	5.24	12	60	12	-	-	-	0.791	0.791	4.82	4.82
Roridin E	0.569	1	5	1	-	-	-	-	0.007	0.141	0.163
**Zearalenones**											
Zearalenone	5.14	17	85	16	1	5.33	-	0.601	0.837	4.68	4.71
Zearalenone-14-Sulfate	2.58	7	35	7	-	-	-	-	1.01	1.01	3.34
**Tropane alkaloids**											
Atropine	0.515	5	25	5	-	-	-	-	0.086	0.086	0.500
Scopolamine	0.515	1	5	1	-	-	-	-	0.007	0.131	0.148

All quantified levels were corrected for recovery. ^$^ Parameters calculated from quantifiable samples only.

**Table 3:** Occurrence of mycotoxins and plant toxins in the analyzed soy and pea protein samples from Germany (only validated analytes with at least one sample > LOD are shown).

	Soy Protein (n = 20)										
Analyte	LOQ [µg/kg]	Positive Samples (>LOD)		# Samples >LOD & <LOQ	# Samples > LOQ	Max ^$^ [µg/kg]	Range ^$^ [µg/kg]	Lower Bound [µg/kg]		Upper Bound [µg/kg]	
		#	Rate (%)					Median	95th Percentile	Median	95th Percentile
**Aflatoxins**											
Aflatoxin B1	1.29	4	20	3	1	1.98	-	-	0.528	0.451	1.73
Aflatoxin G1	0.513	1	5	-	1	2.46	-	-	0.123	0.269	0.379
Aflatoxins (sum of B1, B2, G1, G2)	0.531–1.29	4	20	2	2	2.46	1.98–2.46	-	2.02	1.06	2.69
Sterigmatocystin	0.531	1	5	1	-	-	-	-	0.010	0.210	0.229
***Alternaria*** **toxins**											
Tentoxin	5.17	1	5	1	-	-	-	-	0.089	1.77	1.94
**Citrinin and Ochratoxin A**											
Citrinin	5.15	1	5	1	-	-	-	-	0.111	2.21	2.38
Ochratoxin A	0.541	16	80	2	14	4.52	0.704–4.52	0.984	2.74	0.984	2.74
**Enniatins and Beauvericin**											
Beauvericin	2.07	10	50	4	6	36.0	2.76–36.0	0.217	13.9	1.21	13.9
Enniatin A	2.06	3	15	3	-	-	-	-	0.313	0.313	2.08
Enniatin A1	2.08	10	50	10	-	-	-	0.135	0.271	1.18	2.09
Enniatin B	2.08	19	95	15	4	3.70	2.18–3.70	0.405	2.46	2.10	2.46
Enniatin B1	2.08	10	50	10	-	-	-	0.188	0.375	1.21	2.05
**Zearalenones**											
Zearalenone	5.14	4	20	4	-	-	-	-	1.07	1.07	4.58
**Tropane alkaloids**											
Atropine	0.515	2	10	1	1	1.18	-	-	0.130	0.075	0.548
Scopolamine	0.515	2	10	2	-	-	-	-	0.132	0.132	0.523
	**Pea Protein (n = 20)**										
**Citrinin and Ochratoxin A**											
Citrinin	5.15	1	5	-	1	5.66	-	-	0.283	2.03	2.21
Ochratoxin A	0.541	12	60	3	9	17.0	0.677–17.0	0.149	2.99	0.539	2.99
**Enniatins**											
Enniatin A1	2.08	1	5	1	-	-	-	-	0.014	0.277	0.370
Enniatin B	2.08	6	30	5	1	4.50	-	-	0.608	0.403	2.22
Enniatin B1	2.08	5	25	5	-	-	-	-	0.374	0.374	2.05

All quantified levels were corrected for recovery. ^$^ Parameters calculated from quantifiable samples only.

## Data Availability

The original contributions presented in this study are included in the article/Supplementary Materials. Further inquiries can be directed to the corresponding author.

## Supplementary Materials

The following supporting information can be downloaded at: https://www.mdpi.com/article/10.3390/toxins18090377/s1.

## Abbreviations

The following abbreviations are used in this manuscript:

3Ac-DON	3-Acetyl-Deoxynivalenol
15Ac-DON	15-Acetyl-Deoxynivalenol
ACN	Acetonitrile
AFB1	Aflatoxin B1
AFG1	Aflatoxin G1
Aflas	Aflatoxins
ALTP	Alterperylenol
AME	Alternariolmonomethylether
AME-3S	Alternariolmonomethylether-3-Sulfate
AOH	Alternariol
BEA	Beauvericin
DON	Deoxynivalenol
DON-3G	Deoxynivalenol-3-Glucoside
ENNA	Enniatin A
ENNA1	Enniatin A1
ENNB	Enniatin B
ENNB1	Enniatin B1
EU	European Union
FA	Formic Acid
FB1	Fumonisin B1
FB2	Fumonisin B2
HFB	Hydrolyzed Fumonisin B1
HPLC	High Performance Liquid Chromatography
IS	Internal Standard
LC-MS/MS	Liquid Chromatography–Tandem Mass Spectrometry
LOD	Limit of Detection
LOQ	Limit of Quantification
LVL	Lowest Validated Level
MeOH	Methanol
ML	Maximum Level
MMS	Matrix-Matched Standard
MRM	Multiple Reaction Monitoring
NIV	Nivalenol
OTA	Ochratoxin A
QC	Quality Control
QuEChERS	Quick, Easy, Cheap, Efficient, Rugged, Safe
RSDwR	Within-Laboratory Reproducibility
STC	Sterigmatocystin
TEA	Tenuazonic Acid
TEN	Tentoxin
UPLC	Ultra Performance Liquid Chromatography
ZEN	Zearalenone
ZEN-14S	ZEN-14-Sulfate
ZENs	Zearalenone (ZEN) and related compounds
