# Supplementary material for: Analysis of 113 Mycotoxins and Plant Toxins in Wheat, Soy and Pea Protein Concentrates from the German Market Reveals, i.a., a High Prevalence of Alterperylenol in Wheat Gluten
Source: Toxins (Basel). 2026 Sep 2;18(9):377. doi: 10.3390/toxins18090377 (PMC13611222; doi:10.3390/toxins18090377)
Supplement: Supplementary file 1 [file toxins-18-00377-s001.zip › toxins-4502599-supplementary.pdf]

Supplementary Materials: Analysis of 113 Mycotoxins and Plant Toxins in Wheat, Soy and Pea Protein Concentrates from the German Market Reveals, i.a., a High Prevalence of Alterperylenol in Wheat Gluten

Ahmed H. El-Khatib , Arnold Bahlmann, Christoph Hutzler and Stefan Weigel

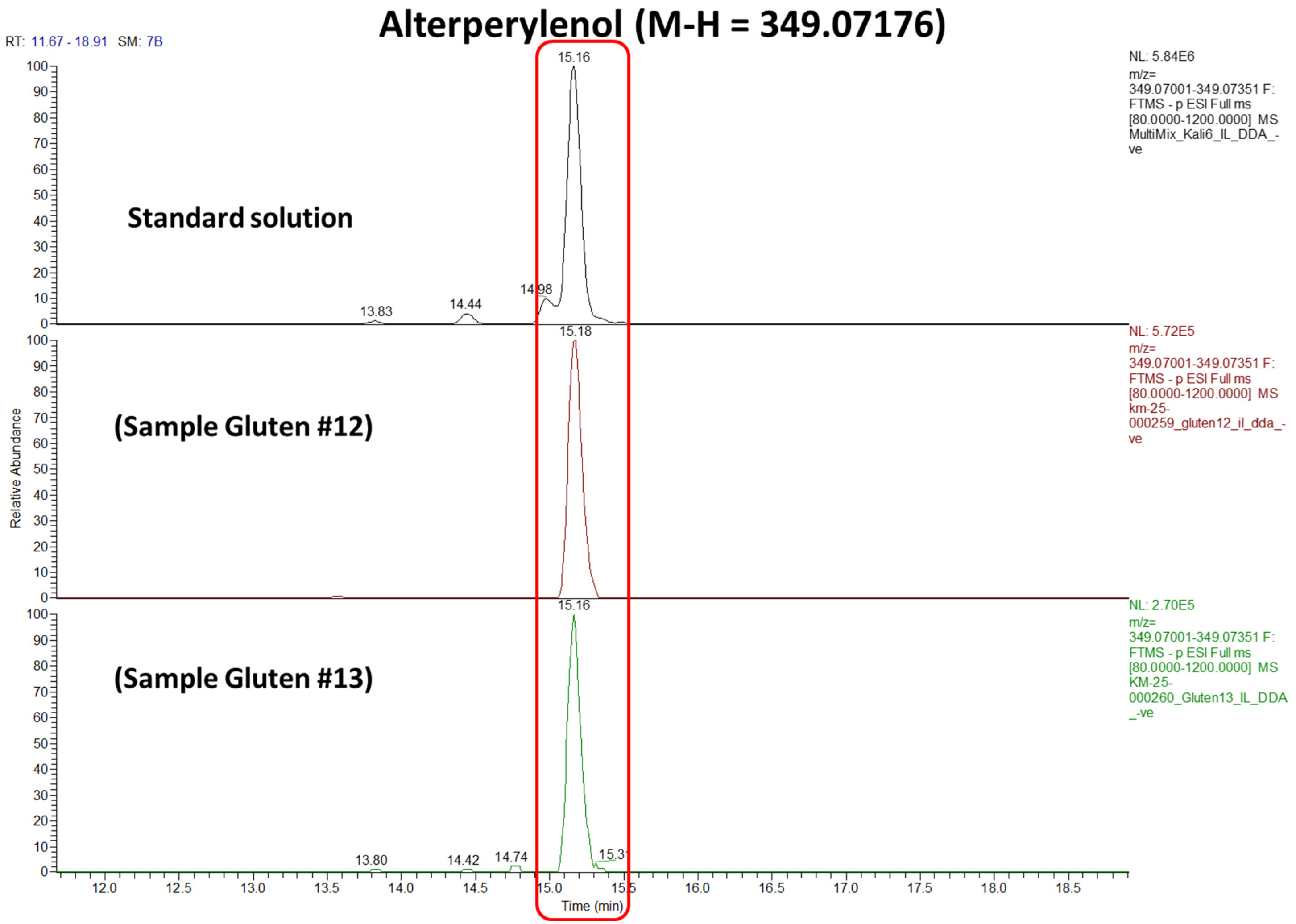

Figure S1a. LC-HR-MS identification of alterperylenol. The extracted ion chromatograms of alterperylenol in standard solution and suspect samples.

Alterperylenol (-ve mode)

MS^2 Comparison

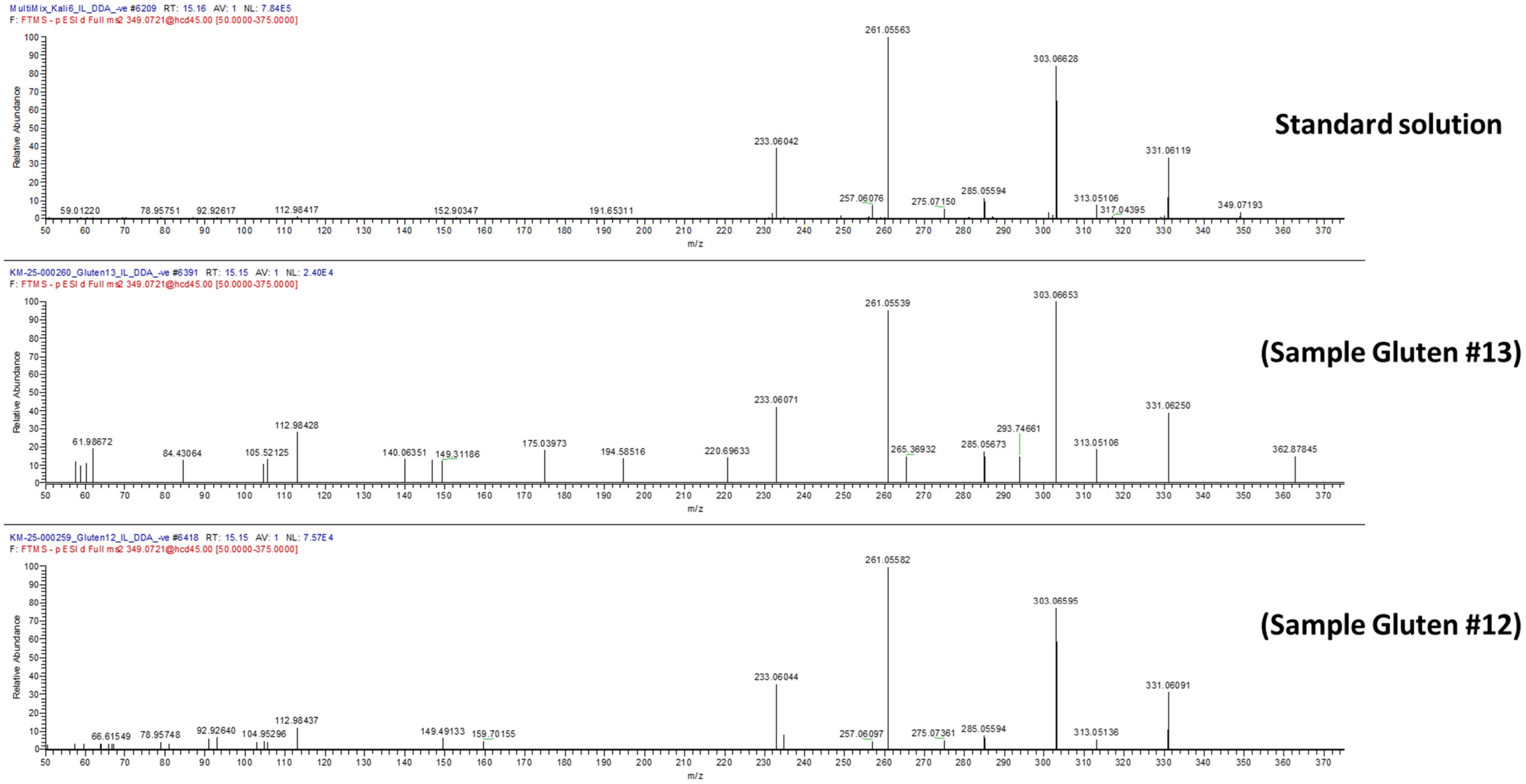

Figure S1b. LC-HR-MS identification of alterperylenol. The MS^2 spectra of alterperylenol in standard solution and suspect samples.

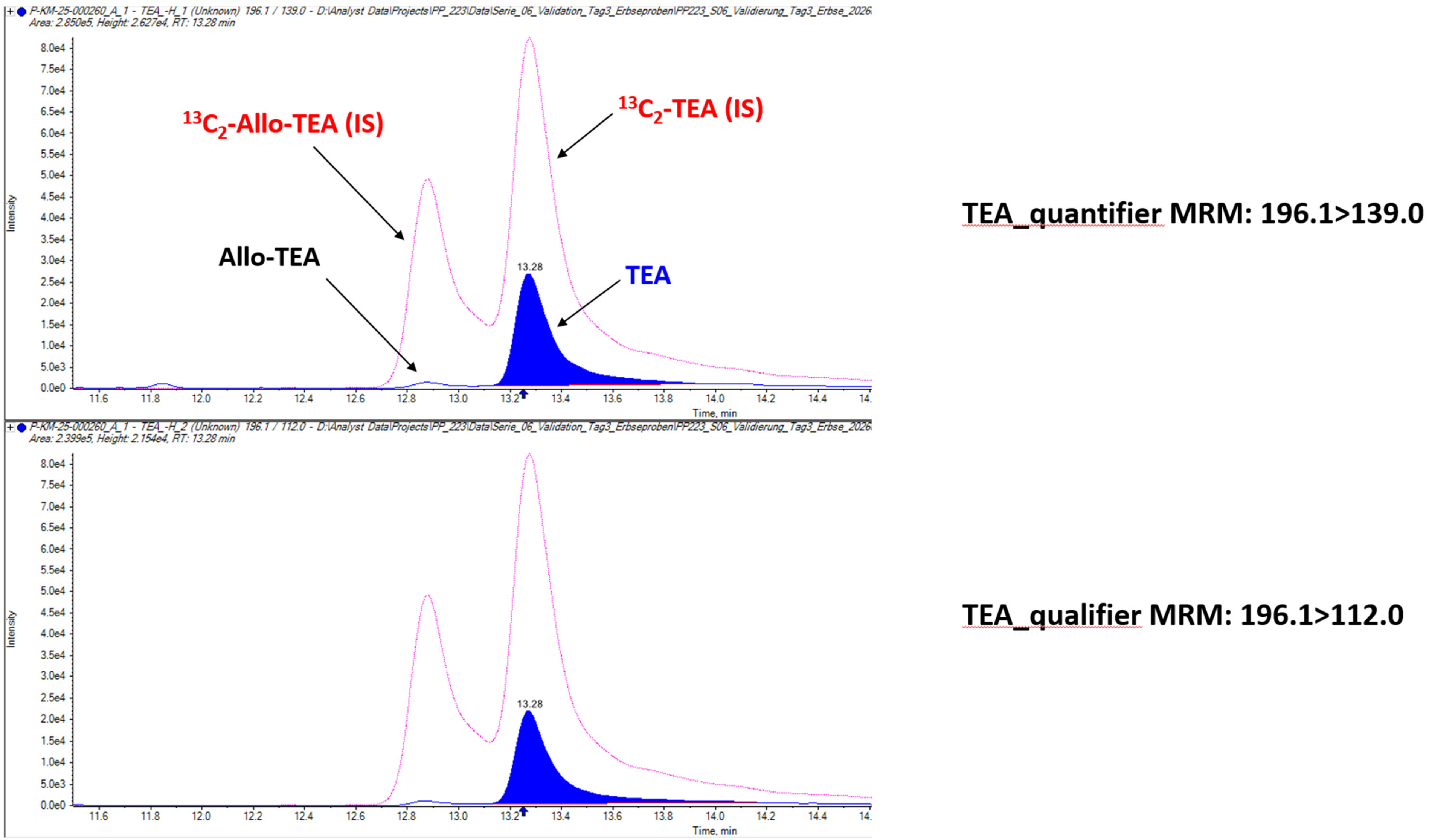

Figure S2. LC-MS/MS signals of tenuazonic acid (TEA) and its isomer allo-TEA in wheat gluten
